# Accuracy and Reliability of the Kinect Version 2 for Clinical Measurement of Motor Function

**DOI:** 10.1371/journal.pone.0166532

**Published:** 2016-11-18

**Authors:** Karen Otte, Bastian Kayser, Sebastian Mansow-Model, Julius Verrel, Friedemann Paul, Alexander U. Brandt, Tanja Schmitz-Hübsch

**Affiliations:** 1 Motognosis UG (haftungsbeschränkt), Berlin, Germany; 2 Center for Lifespan Psychology, Max Planck Institute for Human Development, Berlin, Germany; 3 NeuroCure Clinical Research Center and Clinical and Experimental Multiple Sclerosis Research Center, Charité – Universitätsmedizin Berlin, Berlin, Germany; 4 Department of Neurology, Charité – Universitätsmedizin Berlin, Berlin, Germany; 5 Experimental and Clinical Research Center, Max Delbrück Center for Molecular Medicine and Charité – Universitätsmedizin Berlin, Berlin, Germany; Universitair Medisch Centrum Groningen, NETHERLANDS

## Abstract

**Background:**

The introduction of low cost optical 3D motion tracking sensors provides new options for effective quantification of motor dysfunction.

**Objective:**

The present study aimed to evaluate the Kinect V2 sensor against a gold standard motion capture system with respect to accuracy of tracked landmark movements and accuracy and repeatability of derived clinical parameters.

**Methods:**

Nineteen healthy subjects were concurrently recorded with a Kinect V2 sensor and an optical motion tracking system (Vicon). Six different movement tasks were recorded with 3D full-body kinematics from both systems. Tasks included walking in different conditions, balance and adaptive postural control. After temporal and spatial alignment, agreement of movements signals was described by Pearson’s correlation coefficient and signal to noise ratios per dimension. From these movement signals, 45 clinical parameters were calculated, including ranges of motions, torso sway, movement velocities and cadence. Accuracy of parameters was described as absolute agreement, consistency agreement and limits of agreement. Intra-session reliability of 3 to 5 measurement repetitions was described as repeatability coefficient and standard error of measurement for each system.

**Results:**

Accuracy of Kinect V2 landmark movements was moderate to excellent and depended on movement dimension, landmark location and performed task. Signal to noise ratio provided information about Kinect V2 landmark stability and indicated larger noise behaviour in feet and ankles. Most of the derived clinical parameters showed good to excellent absolute agreement (30 parameters showed ICC(3,1) > 0.7) and consistency (38 parameters showed *r* > 0.7) between both systems.

**Conclusion:**

Given that this system is low-cost, portable and does not require any sensors to be attached to the body, it could provide numerous advantages when compared to established marker- or wearable sensor based system. The Kinect V2 has the potential to be used as a reliable and valid clinical measurement tool.

## Introduction

Kinematic movement analysis has contributed valuable insights into the physiology of movement coordination. It is also used to describe specific impairments of motor function in detail and thus augments clinical diagnosis. As an objective, quantitative technique, some applications have claimed to track changes in motor functions over time more accurately than clinical ratings [1]. This development could lead to the feasibility of clinical ratings based on affordable measurement solutions that do not require trained staff, and thus may be applied outside of the clinical setting [2]. Information on the accuracy of the methods used by these solutions is one fundamental prerequisite for their clinical application. Kinematic movement analysis is most often based on spatiotemporal data of defined anatomic locations. For clinical applications, these are usually transformed into clinically meaningful and interpretable parameters, such as gait speed, range of limb movements and amount of body sway during stance. Here, we explore the suitability of a commercially available motion sensor for clinical movement analysis.

Since the release of the first version of the Kinect sensor in November 2010, this markerless tool has been used in different research scenarios as a low cost alternative to time-of-flight sensors (e.g. SR-4000 CW10 by Mesa technologies) and motion tracking systems (e.g. Vicon, Optotrac). The second generation Kinect (Kinect V2), released in September 2014, is an RGB-Depth (RGB-D) sensor that emits a grid of infrared light. The distance of objects within the camera’s recording range is calculated from time-of-flight analysis of reflected light beams, which yields a depth model of surrounding structures. Based on machine learning techniques, the software development kit (SDK) of the Kinect V2 detects human shapes (up to six people at once). It further provides an artificial skeleton based on 25 artificial anatomical landmarks (‘Kinect joints’) projected into these shapes based on depth data. The sensor improves on the V1 in several respects: it provides depth data with higher spatial resolution, an increased measurement range from 0.8–4m to 0.5–4.5m and increased number of tracked landmarks from 21 to 25. Kinect V1 and V2 have been proposed for the quantification of motor symptoms, for example in posturography [3, 4], gait analysis [5–7] and quantification of hypokinesia in Parkinson’s disease [2]. Gait analysis with multi-camera [8] or single-camera setup [5, 6] in healthy subjects suggested high accuracy for gait speed, stride length, stride time but lower accuracy for other parameters like stride width or speed variability. The accuracy of functional movement parameters of standing balance seemed to depend on observed movement amplitude [3, 4]. However, analysis was confined to trunk landmarks in these studies. Comprehensive studies on Kinect V2 landmark movement accuracy [8, 9] pointed to differences in signal accuracy with landmark location and the direction of movements performed. Importantly, retest reliability was not generally lower with Kinect compared to other motion tracking systems. In addition, investigations of functional movement parameters in a patient cohort found similar accuracy in both Parkinson’s disease and healthy subjects with Kinect V1 [2].

With the present study, we aim to provide a comprehensive evaluation of Kinect V2 accuracy for further development of Kinect V2 into a clinically applicable tool for movement analysis. We first explored the spatiotemporal accuracy of 21 out of 25 different Kinect V2 anatomical landmarks against multi-camera optical motion capture (Vicon) in a set of six motor tasks concerned with balance and lower limb function. Secondly, based on both capturing methods, we analysed the agreement of 45 clinical parameters derived from these tasks and compared their precision in three to five test repetitions. We further propose pre-processing procedures for Kinect V2 data.

## Materials and Methods

### Subjects

Nineteen healthy individuals (age: 29.5 ± 4.4 years, height: 171.7 ± 7.4 cm, 12 female/ 7 male) volunteered to participate in this study. Inclusion criteria were absence of any neurological, motor or cognitive impairment. All participants attended one test session and no inter-day repeated measurements were performed. The study was approved by the Human Research Ethics Committee of Max Planck Institute for Human Development. All subjects provided written informed consent.

### Data Acquisition

**Fig 1:**
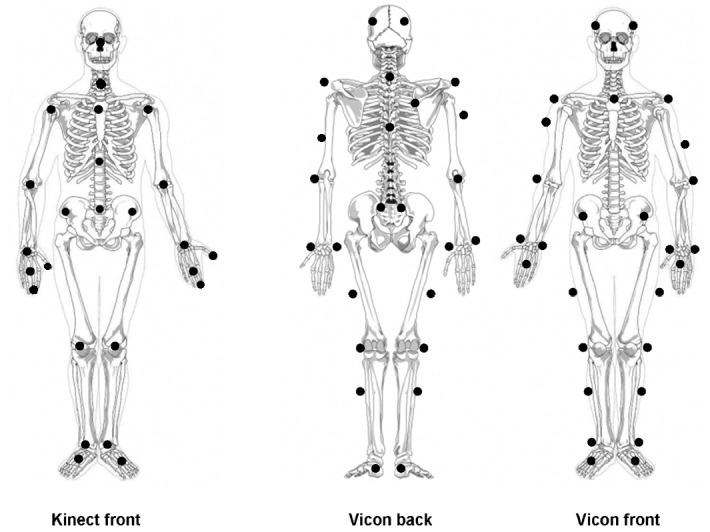
Illustration of marker locations for Kinect skeleton model and Vicon gait model. Adapted from [2] under Creative Common license.

Kinect data were captured with Motognosis Labs v1.0 (Motognosis UG, Berlin, Germany) with a Kinect for Windows V2 Sensor [10] at 30Hz sampling rate. Motognosis Labs used the Software Developer Kit Version 1409 provided by Microsoft [10]. The Kinect sensor was placed on a tripod at 1.4m height with a vertical angle of −8°. The sensor was placed to approximately match the orientation of the coordinate system from the gold-standard reference, facing the frontal plane of the test subjects in all tasks. The Kinect skeleton model with its 25 anatomical landmark locations is shown in Fig 1 (left). As gold-standard reference motion tracking system, we used a 16-camera Vicon system (MX13+, Nexus 2.1; Vicon Motion Systems Ltd., Oxford, UK) using 36 attached IR reflecting markers (Fig 1, middle and right). It was configured to measure marker positions at 100 Hz with 2mm accuracy within an area of 3m by 6m. The Kinect system covered a trapezoid measurement area of roughly 3m by 4m, with a maximum distance of 4.5m to the sensor. Descriptions of all six performed tasks are given in Table 1. All tasks were recorded simultaneously with both systems. The systems were directly connected from Kinect audio output to Vicon’s audio input via cable. Audio start and stop signals were given for offline temporal synchronization. Each task was performed three to five times before measuring the next. For all tasks except walks, patients started at 2.5m distance to the Kinect sensor for best depth resolution [10]. To cover full gait cycles in gait tasks, starting position for these tasks was in 5m distance to the Kinect sensor, which was slightly outside of the sensor range.

**Table 1:** Overview of performed tasks.

Task name	Instruction	Movement Signals	Derived clinical Parameter	Number of Repetitions
Stand up and Sit down (SAS)	After audio signal, stand up and wait for second audio signal, then sit down.	Upper body deviation (movement of shoulder centre relative to spine base), hand range of motion in AP direction	Time needed for stand up and sit down [s], deflection range of upper body during transitions [m], range of motion of each hand in AP direction [m]	5
Short Comfortable speed walk (SCSW)	After audio signal, walk directly towards the sensor at comfortable speed.	Spatial body movement (spine base movement), upper body deviation (movement of shoulder centre relative to spine base), deviation of spine base	Mean speed [m/s], deflection range [°] and mean absolute angular sway velocity [°/s] of upper body sway in pitch and roll direction, left-right and up-down deviation [cm]	5
Short Maximum speed walk (SMSW)	After audio signal, walk directly towards the sensor at maximum speed.	see SCSW	see SCSW	5
Short Line walk (SLW)	After audio signal, walk on an imaginary line directly towards the sensor with heel touching the toes.	see SCSW	see SCSW	5
Stance with closed feet and open and closed eyes (POCO)	Stand with closed feet and open eyes for 20 sec. After audio signal, close eyes for another 20 sec.	Body sway (movement of spine base relative to closed feet position)	pitch, roll and 3D mean absolute angular sway speed [°/s], pitch, roll and 3D sway deflection range [°]	5
Walking on the spot (STEPO)	Walk on the spot at comfortable pace for 40 sec.	AP-V displacement of the knees	Step Frequency (Cadence), knee Range of Motion in AP-V plane[m]	3

Abbrev: AP—anterio-posterior; V—vertical

### Data Processing

Kinematic data acquired with the Vicon system was pre-processed using standard pipelines of the Vicon Software (Nexus 2.1) which includes reconstruction of the model and labeling of the markers, based on individually calibrated subject models. Details on Vicon preprocessing procedures and configuration are provided in the S1 File. Resulting marker labels and marker selection were manually corrected if necessary. Kinect data were used as provided by Kinect SDK without additional pre-processing steps.

Data processing was performed in MATLAB v2015a (MathWorks Inc., Natick, Massachusetts, United States). To compare the movement signals of both systems, spatial and temporal alignment of Vicon and Kinect data were necessary. The following steps were performed for each pair of recordings:

1. The Vicon marker positions were aggregated to represent landmark positions similar to the Kinect skeleton. This was achieved by using the nearest marker of similar representation (e.g. Shoulder, Hand) or by using the mean of the representing markers (e.g. Wrist, Hip). For more detail see S1 Appendix. Since there were no markers for fingertips and thumb in the Vicon marker model, these landmarks of the Kinect model were excluded from further analysis. The resulting mapped skeleton contains therefore only 21 anatomical landmarks.
2. For the alignment of both coordinate systems (origin and axis), we assumed Vicon data (as gold standard) to contain minimal rotation or tilt bias, as this would be corrected during standard pre-processing. Kinect sensor tilts were corrected by using floor normal vectors provided by the Kinect SDK using 3D rotation correction. The final correction step was performed by translation of the Vicon coordinate system to minimize the mean spatial differences between both recordings.
3. Missing values within Vicon data with a gap size smaller than 5 frames were reconstructed by linear interpolation and subsequent re-sampling to 30Hz. No gaps larger than 5 frames were found within all Vicon measurements. Since 5 frames with 100Hz sampling rate are about 1.5 frames in 30Hz, this Vicon interpolation is considered negligible and does not alter the signal behaviour.
4. Due to the loss of audio signals, synchronization by using them resulted in unstable signal offsets. Instead, we used cross correlation shifts of selected landmark movements (see Mentiplay et al. [5]). Respective landmarks were selected for their magnitude of movement depending on the motor task. Since cross correlation requires stationary linear signals [11], the approach was not suitable for gait tasks due to the non-stationary signal in anterior-posterior (AP) dimension. Aligned results still contained temporal offsets of more than 10 frames. Synchronisation for these tasks was therefore achieved with a distance minimization approach based on the AP-signals.

Since one aim of this study was to analyse the accuracy of Kinect landmark movements, these were not smoothed during the data processing steps. The skeleton mapping as performed here (see processing pt. 1) may lead to a spatial bias between corresponding markers and landmarks from both systems. As some analyses require metrical comparison of landmark movements from Kinect and Vicon, we minimized their bias by subtracting the mean of each signal from the signal. This type of signal is further called ‘zero-mean-shifted’.

The processing steps resulted in two 3D skeleton movements with 21 different landmarks sampled at 30Hz. The movements of a single landmark can be described as time series (signals) for each movement dimension. We refer to these signals further on as ‘movement signals’. The movement dimensions are anterio-posterior (AP), medio-lateral (ML) and vertical (V).

### Data Analysis of Movement Signals

Movement signals are the foundation of all kinematic parameters used for movement description. We therefore first analysed the accuracy of movement signals before proceeding to the analysis of derived clinical parameters. The accuracy of movement signals was expressed as the mean 3D Euclidean distance (diff3D) of the zero-mean-shifted movement signals of the Vicon and the Kinect systems and as Pearson’s correlation coefficient (*r*) of each anatomical landmark in each dimension. Based on the thresholds provided by Portney and Watkins, we distinguish between poor (*r* < .4), moderate (*r* = .4 - .7), good (*r* = .7 - 0.9) and excellent (*r* > .9) accuracy [12].

To quantify noise behaviour of Kinect in comparison to the gold standard system, we utilized the signal to noise ratios (SNR) based on the signal variance (see Formula 1) [13].

$$\text{SNR}=10\;{\text{log}}_{10}\left(\frac{var(vicon)}{var(kinect-vicon)}\right)$$ (1)

We assumed movement signals from Vicon to represent the true signal (gold standard) and thus referred to the difference between the zero-mean shifted signals as noise. SNRs were calculated for each landmark and dimension as the ratio between variance of the Vicon signal and variance of the noise. Since SNR is typically given in decibel (dB) a transformation of 10 log 10 was applied [13].

A SNR below 0dB indicates that variance of the noise is larger than the variance of the signal, while 10dB indicates that the signal variance is 10 times larger than the variance of the noise. Since no thresholds for these movement signals are given in literature, we propose thresholds of -10dB and 10dB after visual analysis of the movement signals. Signals with SNR above 10dB can be seen as accurate enough for further analysis. Signals showing SNR below -10dB should be handled with care and are altered or influenced by large noise. Signals that show SNR between -10dB and 10dB seem to be often influenced by small noise or small systematic bias (e.g. in signal amplitude) and should be analysed individually for their suitability of further analyses.

### Outlier Detection

With both motion tracking systems, unreliable landmarks or marker locations may incidentally occur, for instance due to the coverage of landmarks or markers by other body parts. In this case, a ‘jumping’ behaviour of movement signals in one or all dimensions is observed (see S2 and S3 Figs for examples), further called ‘calibration error’. Such calibration errors generally reduce the accuracy of a movement signal. While small, low frequent calibration errors only introduce noise to the signal, large or highly frequent calibration errors can alter a movement signal significantly and would lead to measurement error in derived clinical parameters.

To identify large-amplitude calibration errors that could lead to measurement error, we performed outlier detection prior to the calculation of clinical parameters. Since SNR depends on the signal amplitude, generalised thresholds seemed inappropriate for outlier detection. Based on previous test recordings, we chose Spine base and ankle landmarks as indicators for the occurrence of calibration errors. We derived the maximum velocity and the largest difference of the signal amplitudes between both systems for these landmarks. Based on the limitations of natural movement behaviour, we set lower thresholds of 0.006m/frame for maximum velocity and 0.1m for amplitude differences. If a measurement exceeded both thresholds for one of these landmarks, it was defined as erroneous and excluded from the analyses of derived clinical parameters. However, detected outliers remained in the dataset for the analyses of the movement signal accuracy.

### Extraction and Analysis of clinical Parameters

All tasks targeted different movement behaviours aiming to detect and describe specific motor problems. This necessitated task specific parameter extraction (Table 1). For the ‘stand up and sit down” (SAS) task, postural transition was identified by movement analysis of the shoulder spine landmark and given as time per movement phase and trunk excursion in AP and ML dimension. Additionally, the hand range of motion in AP dimension was calculated as a possible compensatory movement strategy. For all three walk tasks (SCSW, SMSW and SLW), we focused on overall walking speed and quantification of upper body motion during walking as a potential measure of dynamic balance [6, 14]. Due to the short distance, we did not include gait cycle detection and associated parameters like step length and width from our parameter set.

For stance with open and closed eyes (POCO), we analysed body sway at the level of the hip, i.e. close to the body’s centre of gravity as described previously [4]. For walking on the spot (STEPO), we focused on the quantification of lower limb movements described by ranges of motion (RoM) on anterio-posterior-vertical (AP-V) plane and step count per minute (cadence) as a potential measure of for instance muscular weakness, hypokinesia or muscle fatiguing.

In total, 45 different clinical parameters were extracted.

### Statistical Analysis

Statistical analysis was performed in MATLAB v2015a (MathWorks Inc., Natick, Massachusetts, United States) and visualised in Python 3.4 using the packages ‘seaborn’ and ‘matplotlib’. ICC(1,1) (one-way random model) and standard error of measurement (SEM) were used to describe repeatability of derived parameters for each system. For better comparison of parameters, the SEM was expressed as proportion of the mean. Absolute agreement between Vicon and Kinect was described by ICC(3,1) (two-way mixed model) and limits of agreement (LOA). Pearson’s correlation coefficient (*r*) was used to describe consistency by neglecting systematic measurement bias.

## Results

### Accuracy of Movement Signals

**Fig 2:**
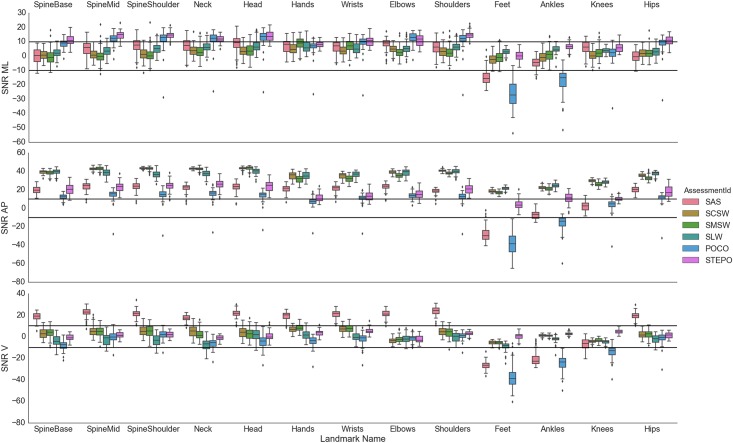
Signal-to-Noise ratios (SNR) of all joints per assessment in each dimension. Bi-lateral joints were aggregated by their mean for better visualisation. Abbrev: AP—anterio-posterior; ML—medio-lateral; V—vertical.

Spatial accuracy of the Kinect landmark movements is reported as 1) mean Euclidean 3D distances (diff3D) between temporally aligned zero-mean shifted signals to show absolute differences for signal pairs and 2) Pearson’s correlation coefficients (*r*) against Vicon markers. In addition, signal to noise ratios (SNR) are reported in AP, ML and V dimension each. As an overview, means and standard deviations for all expressions of signal accuracy averaged over all tasks, subjects and measurement repetitions are shown in Table 2. Task specific landmark accuracy is provided in Fig 2 and S2 Appendix.

**Table 2:** Accuracy of movement signals from Kinect landmarks against Vicon marker locations expressed as mean 3D Euclidian distance diff3D, Pearson correlation coefficients *r* per dimension and signal-to-noise ratio SNR per dimension. Data are presented as mean and standard deviation (SD) of all measurements (including all subjects, tasks and measurement repetitions).

Joint name	diff_3D_ [m]	*r*_*AP*_	*r*_*ML*_	*r*_*V*_	SNR_AP_	SNR_ML_	SNR_V_
Head	0.01 (0.01)	0.99 (0.05)	0.90 (0.17)	0.73 (0.30)	31.69 (12.41)	7.90 (5.98)	4.70 (9.84)
Neck	0.02 (0.01)	0.99 (0.05)	0.88 (0.16)	0.46 (0.52)	31.37 (11.47)	6.96 (5.40)	1.87 (9.56)
Spine shoulder	0.01 (0.01)	0.99 (0.05)	0.86 (0.23)	0.68 (0.37)	31.30 (11.78)	6.19 (6.72)	5.70 (9.07)
Spine mid	0.01 (0.01)	0.99 (0.05)	0.85 (0.23)	0.70 (0.36)	31.48 (11.89)	5.51 (6.61)	5.43 (9.54)
Spine base	0.02 (0.01)	0.99 (0.02)	0.81 (0.24)	0.66 (0.35)	28.80 (11.78)	3.24 (5.73)	2.26 (9.69)
Left shoulder	0.01 (0.01)	0.99 (0.05)	0.87 (0.20)	0.74 (0.28)	29.03 (12.03)	6.61 (6.39)	6.63 (9.06)
Left elbow	0.02 (0.01)	0.99 (0.02)	0.89 (0.16)	0.46 (0.39)	28.31 (10.83)	7.39 (5.19)	1.17 (10.07)
Left wrist	0.02 (0.01)	0.98 (0.05)	0.89 (0.15)	0.80 (0.25)	25.53 (10.74)	7.30 (4.87)	7.06 (8.39)
Left hand	0.03 (0.02)	0.97 (0.07)	0.89 (0.13)	0.76 (0.27)	23.91 (11.70)	7.14 (4.54)	6.44 (8.32)
Right shoulder	0.01 (0.01)	0.99 (0.05)	0.89 (0.17)	0.72 (0.27)	28.76 (12.37)	7.13 (5.95)	5.87 (9.57)
Right elbow	0.02 (0.01)	0.99 (0.05)	0.87 (0.18)	0.51 (0.37)	28.54 (11.32)	6.89 (5.88)	2.56 (9.56)
Right wrist	0.02 (0.02)	0.98 (0.06)	0.86 (0.18)	0.78 (0.25)	25.98 (11.12)	6.34 (5.51)	6.07 (8.86)
Right hand	0.02 (0.02)	0.97 (0.07)	0.86 (0.17)	0.74 (0.29)	24.83 (12.24)	6.54 (5.25)	5.77 (8.58)
Left hip	0.02 (0.01)	0.99 (0.05)	0.82 (0.22)	0.61 (0.35)	26.53 (10.91)	4.06 (5.44)	3.41 (8.41)
Left knee	0.03 (0.02)	0.87 (0.28)	0.78 (0.20)	0.19 (0.41)	17.28 (12.60)	2.83 (4.65)	-5.35 (6.38)
Left ankle	0.05 (0.03)	0.78 (0.35)	0.64 (0.33)	0.29 (0.41)	9.73 (17.71)	-2.46 (9.34)	-7.27 (11.87)
Left foot	0.06 (0.04)	0.64 (0.46)	0.47 (0.35)	-0.02 (0.37)	-0.47 (25.15)	-7.25 (11.48)	-14.30 (14.48)
Right hip	0.02 (0.01)	0.99 (0.05)	0.81 (0.25)	0.64 (0.34)	26.16 (10.68)	3.97 (5.60)	3.99 (8.36)
Right knee	0.04 (0.02)	0.87 (0.28)	0.80 (0.21)	0.19 (0.41)	16.93 (12.64)	3.60 (5.09)	-5.42 (6.33)
Right ankle	0.05 (0.04)	0.81 (0.33)	0.64 (0.32)	0.22 (0.38)	8.63 (16.27)	-2.26 (10.25)	-8.33 (12.49)
Right foot	0.07 (0.04)	0.66 (0.44)	0.49 (0.36)	-0.03 (0.35)	-1.94 (26.03)	-7.83 (13.17)	-15.83 (14.96)

*r* refers to the pearson correlation coefficient. Results are shown as mean values of all assessments, measurement repetitions and subjects. Abbrev: AP—anterio-posterior; ML—medio-lateral; V—vertical

The 3D differences between Vicon and Kinect V2 movement signals were typically between 1 and 2cm, except for higher values for feet and ankles (diff3D > 5cm). Pearson’s correlation coefficients (*r*) were highest in AP dimension and good (4) or excellent (15) in all landmarks with excellent spatial agreement. Exceptionally, only moderate correlations were seen for feet. Signals in ML dimension provided good results as well (head excellent, 16 landmarks good, feet and ankle moderate). However, in vertical dimension, correlations were only poor (6) or moderate (7) to good (8). Observed accuracy also varied with landmark location, with head having the highest and feet the lowest values (*r*_*AP*_ foot L/R = 0.64/0.66; *r*_*AP*_ head = 0.99). Furthermore, spatial accuracy of landmarks was found to depend on the measured task, for example the Spine base landmark in quiet stance (POCO) had *r*_*ML*_ = 0.95, but *r*_*ML*_ = 0.64 in stand up and sit down (SAS) (see S2 Appendix).

Fig 2 presents SNR per task, landmark and dimension as an indicator of overall signal quality. The standard deviations of SNR are smaller than those of the correlation analyses (see S1 Fig) making SNR results easier to interpret. Similar to the Pearson’s correlation coefficients (*r*), the most robust signal quality, i.e. the highest SNRs, are in AP dimension (most > 10dB). SNRs in ML dimension were smaller and less consistent between different tasks (most upper body landmarks > 5dB). The lowest SNRs were seen in V dimension (most between -10db and 8dB) with the exception of SAS, that showed large SNR (> 18dB) in V dimension, probably related to the large vertical movement in this task. Feet and ankles generally showed small SNR in all dimensions (SNR < 0dB), especially in SAS and POCO tasks, i.e. with feet stable on the ground throughout the task. The best mean SNRs for feet in V dimension for STEPO tasks were still near 0dB (Feet SNR V = 0.5dB), while, the ankle and knee landmarks seemed more stable (ankle SNR V = 2.73dB; knee SNR V = 5.71dB).

### Outlier detection

In total, 13 out of 532 measurements were detected that contained large calibration errors and were excluded from the calculation of clinical parameters. These comprised 12 Kinect and 1 Vicon measurements from the following tasks: SAS (1 Kinect and 1 Vicon), SLW (2 Kinect) and POCO (9 Kinect). Calibration errors were most prominent in V dimension (11 measurements) with 3 Measurements showing additional errors in AP dimension. As expected, detected outliers had highly negative mean SNRs of -29,55 dB in V dimension, -40,51 dB in AP and -44,16 dB in ML dimension. An overview of the outliers is given in the S1 Table.

### Accuracy and Repeatability of clinical Parameters

As shown in Table 3, most of the 45 clinical parameters showed good to excellent absolute agreement (ICC(3,1): 30 parameters > 0.7) and consistency (*r*: 38 parameters > 0.7), Absolute agreement was especially high for trunk movement and time needed for postural transitions, gait speed determined from short walks at different speeds, sway velocity in quiet standing, as well as cadence while walking in place. Lower accuracy was determined for roll trunk movement in roll direction in all short walks (ICC(3,1) of 0.43–0.65). For knee RoM in AP-V plane while walking on the spot, low accuracy (ICC(3,1) < 0.12) was accompanied by good consistency (*r*_*L*_ = 0.72, *r*_*R*_ = 0.83) for this parameter. This may be explained by a systematic measurement bias of 0.05m in this parameter. Since a measurement bias was only observed in this parameter, a general, systematic bias between both systems is unlikely. Up-down deviation during short line walk was the only parameter that showed poor absolute agreement (ICC(3,1) = 0.03) and poor consistency agreement (*r* = 0.09). This is likely attributable to the small RoM (ca. 0.4cm) and the noise behaviour of the Spine base in vertical dimension (SNR < 0). This is likely attributable to the small RoM (ca. 0.4cm), the noise behaviour of the hip joints (SNR < 0) and generally poor accuracy for vertical movement components.

**Table 3:** Clinical parameters derived from the six tasks with either kinematic method. Means and standard deviations (SD) are given along with accuracy (ICC(3,1) and Pearson’s *r*, LOA in % of methods’ mean).

	Accuracy					
	Kinect Mean (SD)	Vicon Mean (SD)	Diff	LOA [%]	ICC(3,1)	Pearson’s *r*
*Stand up and Sit down*						
Up Time [s]	1.31 (0.25)	1.38 (0.25)	0.07	6.74	0.95 (0.31; 0.99)	0.98
Up DR ML [cm]	2.42 (1.42)	2.15 (1.16)	-0.26	70.57	0.78 (0.67; 0.86)	0.81
Up DR AP [cm]	37.02 (6.51)	37.22 (6.41)	0.19	5.77	0.99 (0.98; 0.99)	0.99
Up RoM right Hand [cm]	31.74 (6.28)	32.39 (6.34)	0.65	11.69	0.95 (0.92; 0.97)	0.95
Up RoM left Hand [cm]	34.04 (6.96)	34.67 (7.07)	0.62	9.36	0.97 (0.95; 0.98)	0.97
Down Time [s]	1.53 (0.23)	1.59 (0.23)	0.06	9.09	0.92 (0.71; 0.97)	0.95
Down DR ML [cm]	2.87 (1.45)	2.37 (1.26)	-0.49	68.31	0.73 (0.51; 0.84)	0.78
Down DR AP [cm]	39.67 (6.27)	39.29 (5.97)	-0.37	11.38	0.93 (0.89; 0.95)	0.93
*comfortable speed walk*						
Speed Mean [m/s]	1.29 (0.16)	1.28 (0.15)	-0.01	1.34	1.00 (0.99; 1.00)	1.00
Left-right deviation [cm]	1.40 (0.45)	1.19 (0.48)	-0.20	41.94	0.75 (0.41; 0.88)	0.82
Up-down deviation [cm]	0.80 (0.22)	0.77 (0.20)	-0.03	21.76	0.91 (0.85; 0.94)	0.92
DR pitch [°]	5.70 (1.86)	5.20 (1.66)	-0.50	40.77	0.76 (0.61; 0.85)	0.80
DR roll [°]	3.20 (1.14)	3.18 (0.99)	-0.02	70.21	0.43 (0.25; 0.58)	0.43
DR 3D [°]	4.06 (1.44)	5.11 (1.54)	1.05	59.12	0.45 (0.10; 0.67)	0.57
MSV pitch [°/s]	6.11 (1.13)	5.49 (1.61)	-0.62	33.82	0.68 (0.40; 0.81)	0.79
MSV roll [°/s]	4.91 (1.65)	4.67 (1.71)	-0.25	73.03	0.43 (0.25; 0.58)	0.43
MSV 3D [°/s]	8.66 (1.64)	7.91 (1.91)	-0.75	40.03	0.50 (0.30; 0.66)	0.55
*maximum speed walk*						
Speed Mean [m/s]	2.10 (0.21)	2.08 (0.21)	-0.02	0.98	0.99 (0.66; 1.00)	1.00
Left-right deviation [cm]	1.27 (0.38)	0.72 (0.34)	-0.55	53.16	0.33 (-0.08; 0.68)	0.72
Up-down deviation [cm]	0.81 (0.42)	0.88 (0.52)	0.07	47.43	0.90 (0.84; 0.93)	0.93
DR pitch [°]	12.04 (3.22)	13.43 (3.94)	1.39	20.28	0.87 (0.35; 0.95)	0.95
DR roll [°]	4.00 (1.39)	4.88 (1.90)	0.88	69.01	0.49 (0.24; 0.66)	0.58
DR 3D [°]	9.34 (3.41)	13.28 (3.85)	3.94	39.52	0.51 (-0.09; 0.80)	0.81
MSV pitch [°/s]	10.74 (3.46)	11.94 (4.17)	1.19	22.35	0.90 (0.55; 0.96)	0.96
MSV roll [°/s]	8.17 (2.68)	9.17 (3.84)	1.00	59.82	0.65 (0.49; 0.76)	0.72
MSV 3D [°/s]	14.79 (4.12)	16.18 (5.29)	1.38	27.83	0.86 (0.67; 0.93)	0.92
*Line walk*						
Speed Mean [m/s]	0.24 (0.05)	0.24 (0.05)	-0.00	1.02	1.00 (0.92; 1.00)	1.00
Left-right deviation [cm]	2.04 (0.78)	1.87 (0.71)	-0.17	20.58	0.94 (0.76; 0.97)	0.97
Up-down deviation [cm]	0.45 (0.13)	0.32 (0.06)	-0.14	69.30	0.03 (-0.07; 0.16)	0.09
DR pitch [°]	8.99 (2.67)	8.08 (2.81)	-0.91	34.33	0.81 (0.59; 0.90)	0.85
DR roll [°]	7.14 (2.48)	9.90 (4.26)	2.75	56.16	0.58 (-0.01; 0.81)	0.87
DR 3D [°]	8.42 (2.74)	8.48 (3.04)	0.06	45.69	0.77 (0.67; 0.84)	0.77
MSV pitch [°/s]	3.57 (0.65)	3.31 (0.70)	-0.26	31.27	0.62 (0.41; 0.75)	0.67
MSV roll [°/s]	3.53 (0.98)	4.39 (1.38)	0.86	46.96	0.54 (0.07; 0.76)	0.72
MSV 3D [°/s]	5.61 (1.02)	6.02 (1.39)	0.41	29.37	0.71 (0.52; 0.82)	0.78
*Stance with closed feet and open and closed eyes*						
DR pitch [°]	1.67 (0.76)	1.49 (0.63)	-0.17	25.15	0.93 (0.70; 0.97)	0.97
DR Roll [°]	1.54 (0.52)	1.35 (0.40)	-0.19	30.06	0.81 (0.39; 0.92)	0.91
DR 3D [°]	1.60 (0.73)	1.50 (0.60)	-0.11	32.17	0.92 (0.85; 0.95)	0.95
MSV pitch [°/s]	0.17 (0.06)	0.18 (0.06)	0.01	14.24	0.97 (0.93; 0.99)	0.98
MSV roll [°/s]	0.21 (0.07)	0.20 (0.06)	-0.00	23.29	0.93 (0.89; 0.96)	0.94
MSV 3D [°/s]	0.30 (0.10)	0.30 (0.09)	0.00	16.29	0.96 (0.94; 0.98)	0.97
*Walking on the spot*						
RoM Knee L [cm]	4.37 (1.06)	9.05 (1.70)	4.68	34.75	0.10 (-0.03; 0.36)	0.72
RoM Knee R [cm]	4.38 (1.02)	9.03 (1.89)	4.64	34.90	0.12 (-0.03; 0.41)	0.83
Cadence L [steps/min]	47.08 (7.31)	47.00 (7.21)	-0.08	1.40	1.00 (1.00; 1.00)	1.00
Cadence R [steps/min]	47.63 (7.18)	47.58 (7.15)	-0.05	1.14	1.00 (1.00; 1.00)	1.00

Abbrev: AP—anterio-posterior; ML—medio-lateral; V—vertical; LOA—limits of agreement; DR—deflection range; MSV—mean sway velocity; RoM—range of motion.

To address repeatability of each parameter and for each method, ICC(1,1) and Standard Error of Measurement (SEM) as percentage of mean were calculated (see Table 4). ICC(1,1) was acceptable for most parameters (ICC(1,1) > 0.6 Kinect: 33; Vicon:30). More importantly, repeatability results were of similar magnitude for both, Kinect V2 and Vicon derived parameters (ICC(1,1) Kinect V2.20–0.98; Vicon.28–0.98). Relative SEM was acceptable (< 20%) for Kinect V2 in 31 of 45 parameters investigated (Vicon: 30). This included all parameters with high between-method agreement as outlined above. In total, 12 parameters showed good SEM (< 10%) in Vicon and Kinect, including walking speeds in all walk tests, time parameters and AP movement components of SAS and all parameters from STEPO.

**Table 4:** Clinical parameters derived from the six tasks with either kinematic method. Means and standard deviations (SD) are given along with repeatability (ICC(1,1) and SEM in % of mean).

	Repeatability			
	Kinect ICC(1,1)	Kinect SEM [%]	Vicon ICC(1,1)	Vicon SEM [%]
*Stand up and Sit down*				
Up Time [s]	0.72 (0.55; 0.86)	9.89	0.77 (0.62; 0.89)	8.59
Up DR ML [cm]	0.56 (0.35; 0.76)	39.08	0.43 (0.22; 0.67)	40.71
Up DR AP [cm]	0.81 (0.68; 0.91)	7.57	0.82 (0.69; 0.92)	7.25
Up RoM right Hand [cm]	0.73 (0.56; 0.87)	10.24	0.70 (0.52; 0.85)	10.69
Up RoM left Hand [cm]	0.69 (0.50; 0.84)	11.46	0.68 (0.50; 0.84)	11.48
Down Time [s]	0.55 (0.34; 0.76)	9.91	0.62 (0.42; 0.80)	9.10
Down DR ML [cm]	0.49 (0.28; 0.71)	36.41	0.33 (0.13; 0.59)	43.50
Down DR AP [cm]	0.73 (0.56; 0.87)	8.20	0.79 (0.65; 0.90)	6.94
*comfortable speed walk*				
Speed Mean [m/s]	0.81 (0.67; 0.91)	5.38	0.80 (0.67; 0.91)	5.29
Left-right deviation [cm]	0.40 (0.20; 0.64)	25.03	0.49 (0.28; 0.71)	28.75
Up-down deviation [cm]	0.88 (0.79; 0.95)	9.42	0.93 (0.86; 0.97)	7.15
DR pitch [°]	0.32 (0.13; 0.58)	26.79	0.28 (0.10; 0.54)	26.98
DR roll [°]	0.79 (0.65; 0.90)	16.42	0.55 (0.35; 0.75)	20.96
DR 3D [°]	0.45 (0.24; 0.68)	26.34	0.30 (0.11; 0.55)	25.21
MSV pitch [°/s]	0.64 (0.45; 0.81)	11.17	0.83 (0.71; 0.92)	12.18
MSV roll [°/s]	0.81 (0.67; 0.91)	14.69	0.75 (0.59; 0.88)	18.27
MSV 3D [°/s]	0.71 (0.54; 0.86)	10.13	0.81 (0.68; 0.91)	10.50
*maximum speed walk*				
Speed Mean [m/s]	0.86 (0.76; 0.94)	3.72	0.87 (0.77; 0.94)	3.65
Left-right deviation [cm]	0.44 (0.24; 0.68)	22.14	0.33 (0.13; 0.58)	38.75
Up-down deviation [cm]	0.83 (0.71; 0.92)	21.43	0.91 (0.84; 0.96)	17.76
DR pitch [°]	0.60 (0.40; 0.79)	17.00	0.61 (0.42; 0.79)	18.32
DR roll [°]	0.63 (0.44; 0.81)	21.25	0.54 (0.34; 0.75)	26.34
DR 3D [°]	0.67 (0.49; 0.83)	21.06	0.58 (0.39; 0.78)	18.73
MSV pitch [°/s]	0.72 (0.56; 0.86)	16.99	0.74 (0.58; 0.87)	17.80
MSV roll [°/s]	0.77 (0.63; 0.89)	15.58	0.81 (0.68; 0.91)	18.16
MSV 3D [°/s]	0.76 (0.61; 0.88)	13.54	0.85 (0.73; 0.93)	12.88
*Line walk*				
Speed Mean [m/s]	0.92 (0.85; 0.97)	5.78	0.92 (0.86; 0.97)	5.83
Left-right deviation [cm]	0.78 (0.63; 0.90)	17.74	0.71 (0.54; 0.86)	20.25
Up-down deviation [cm]	0.20 (0.03; 0.46)	25.22	0.68 (0.50; 0.84)	10.92
DR pitch [°]	0.65 (0.45; 0.82)	17.66	0.64 (0.44; 0.82)	20.92
DR roll [°]	0.50 (0.29; 0.73)	24.54	0.50 (0.29; 0.72)	30.52
DR 3D [°]	0.66 (0.47; 0.83)	19.09	0.56 (0.35; 0.77)	23.84
MSV pitch [°/s]	0.60 (0.40; 0.79)	11.48	0.65 (0.46; 0.82)	12.51
MSV roll [°/s]	0.67 (0.48; 0.83)	15.95	0.62 (0.42; 0.81)	19.28
MSV 3D [°/s]	0.65 (0.46; 0.82)	10.78	0.66 (0.48; 0.83)	13.40
*Stance with closed feet and open and closed eyes*				
DR pitch [°]	0.45 (0.22; 0.72)	33.95	0.43 (0.20; 0.71)	31.83
DR Roll [°]	0.41 (0.18; 0.69)	25.66	0.36 (0.13; 0.65)	23.67
DR 3D [°]	0.47 (0.23; 0.73)	33.45	0.42 (0.19; 0.70)	30.73
MSV pitch [°/s]	0.75 (0.56; 0.89)	18.05	0.77 (0.59; 0.90)	15.92
MSV roll [°/s]	0.77 (0.60; 0.91)	16.42	0.78 (0.60; 0.91)	14.04
MSV 3D [°/s]	0.82 (0.67; 0.93)	13.99	0.84 (0.70; 0.94)	11.67
*Walking on the spot*				
RoM Knee L [cm]	0.86 (0.73; 0.94)	9.23	0.93 (0.85; 0.97)	5.09
RoM Knee R [cm]	0.93 (0.87; 0.97)	5.94	0.91 (0.82; 0.96)	6.31
Cadence L [steps/min]	0.98 (0.95; 0.99)	2.39	0.98 (0.95; 0.99)	2.36
Cadence R [steps/min]	0.96 (0.92; 0.98)	2.93	0.96 (0.92; 0.98)	2.94

Abbrev: AP—anterio-posterior; ML—medio-lateral; V—vertical; SEM—Standard Error of Measurement; DR—deflection range; MSV—mean sway velocity; RoM—range of motion.

## Discussion

In the present study, we evaluated the suitability of the Kinect V2 sensor for clinical motion analyses against a gold standard reference system, namely Vicon. We analysed landmark movement accuracies as well as the accuracy and reliability of different clinical parameters derived from six motor tasks in young healthy subjects. Caution should be taken since, the presented results can only be generalised for young healthy adults.

### Methods, Setup and Data Processing

The automatically labelled anatomical landmarks from Kinect yielded signals of sufficient calibration accuracy in 520 of 532 measurements compared to 531 Vicon measurements. This is remarkable, as calibration with Vicon in our experience required far more manual processing effort. The aggregation of Vicon markers that was chosen according to Galna et al. seemed appropriate as only minimal spatial offsets were observed between the aligned signals from Kinect V2 and Vicon. However, with the inherent differences between the 3D skeletons of both methods in mind, i.e. surface markers with Vicon versus landmarks within the body shape with Kinect V2, this approach was not intended to achieve exact location matching. As derived clinical parameters are calculated only within each method’s coordinate system without any reference to absolute external locations, this approach may slightly affect 3D Euclidean distances, but is not expected to affect correlation analyses and agreement of clinical parameters. For the same reason, the spatial alignment used here is considered appropriate for the purpose of our study. If anatomical correctness was to be studied (such as in [15–17]), synchronisation should rather use a multi-point minimization approach [9]. The higher Euclidean distances seen for foot landmarks coincide with low between-method correlations and low SNR. We therefore consider the spatial offset for these landmarks not due to differences in skeleton models but attributable to signal noise, for example a higher rate of calibration errors. Concerning temporal synchronisation by audio signals, we expected delays and remaining offsets of < 500ms due to the varying latency in sound card processing [18]. Unexpectedly, synchronization by audio signals turned out to be unreliable due to signal losses from the Kinect to the Vicon system. The synchronisation by cross-correlation and distance minimization seemed reliable, since no detected temporal offsets remained, but required manual selection of suitable landmark movements. For future work, the Network Time Protocol [9] or the Precision Time Protocol [19] seem more appropriate, especially if one system is expected to show temporal delay during recording.

### Movement signal accuracy

Main findings from this part of analysis were the differences in signal accuracy according to 1) directional components (lowest for vertical), 2) landmark location (lowest for feet) and 3) performed movement task. The last is possibly attributable to the differences in movement amplitudes. As one conclusion of this study, 3D positions of axial landmarks (Spine base, Spine mid, Spine shoulder and Head) and upper body extremity landmarks (Hand, Elbow and Shoulder) can validly be used for general movement analyses and calculation of clinical parameters.

Concerning the differences in accuracy between the directional components, our data support previous reports on clinical parameters derived from Kinect V2 trunk landmarks during standing [3], where highest accuracy was also observed for AP compared to ML movements, while V components were not reported. In our study, *r* in V dimension did not exceed 0.8 in any of the landmarks and was < 0.7 in 13 out of 21 landmarks. Interestingly, highest accuracies in the vertical movement components (*r* > 0.7) were observed for head, shoulder, (not elbow), wrist and hand signals. A similar pattern for the accuracy of limb landmarks was seen in a recent study that used a Kinect V2 multi-camera setup [8]. We interpret this as a consequence of Kinect SDK optimization for the intended use of the Kinect sensors in the context of interactive computer-gaming based on gesture recognition. For all other landmarks with low accuracy in the vertical dimension, different recording angles may be explored to increase accuracy, if the tracking of (minor) vertical displacement is of interest.

Signals of feet (and ankles) had the lowest accuracy according to mean 3D distance and correlation analysis in all dimensions. Their low negative SNRs point to a general instability of this landmark location that differs with the task (Fig 2), with the worst SNR for stable foot positions throughout the task. This has also been noted by others [9] and is interpreted as a specific difficulty of Kinect V2 to differentiate feet from ground in such conditions. Furthermore, differences in signal accuracy were seen between tasks for the same landmarks. One explanation is that the accuracy of movement signals is influenced by the respective landmark’s range of motion [3], and increases with larger movements as larger signals favourably alter the SNR. The reason is, that the noise is proportionally smaller in signals with larger amplitudes. This is supported by the high accuracy for AP in walks, and for V in SAS for head, trunk, arms and hips. In terms of noise behaviour, the instability of landmark locations according to SNR are first, reflected in generally lower signal agreement for the same landmarks, and, second, are outweighed by signal increases such that accuracy improves. Other possible factors that may contribute to differences in accuracy are differences in body posture or coverage of landmarks to different extents with different tasks (e.g. feet in SLW task). For clinical applications, we therefore recommend to design movement tasks preferably to not cover landmarks during execution. Nevertheless, as outlined above, advanced filtering techniques or alternative skeleton models may also derive more accurate clinical parameters even for small movements, like tremor, or temporarily covered landmark locations.

### Accuracy and Reliability of clinical Parameters

Based on clinical assessment routines, we extracted 45 different parameters to describe the movement behaviour of each subject. Previous publications showed, that Kinect V1 and V2 measurements of landmark angles [16, 20, 21] and length of body parts [22] derived from different movements may lack accuracy. Therefore, we focused on parameters based on single ‘stable’ landmarks with the exception of POCO, where foot landmarks were integrated into an anchor point for the sway vector.

In summary, most clinical parameters showed high absolute agreement and no systematic bias between systems. The parameters that showed moderate absolute agreement mostly showed high consistency agreement as well. This leads us to the assumption that the Kinect V2 is accurate enough to measure these clinical parameters in healthy subjects. Our data concur with previous reports on gait analysis with Kinect V2 [5] with respect to comfortable and maximum speeds including high accuracy and repeatability for these parameters. Galna et al. used Kinect V1 to analyse a task similar to SAS. Although they measured performance time of 5 stand up-sit down tasks, whereas we assessed both transition phases of the movement separately, the Pearson’s correlation coefficients against the Vicon standard are equally high (Galna et al. *r* = .999 vs *r* = .98 here). The same study also analysed the stepping on the spot task and observed somewhat higher cadence (Galna et al. 50.85 steps/min vs. 47.3 steps/min here) but similar accuracy for this parameter (Galna et al. *r* = 0.983 vs. *r* = 1 here), whereas we found a systematic spatial bias for knee RoM. Further investigation should analyse the cause of this bias and their impact on clinical interpretation.

As discussed above, the dependency of movement signal accuracy on movement amplitude may impact derived clinical parameters. For instance, smaller movement parameters show larger LOAs (see e.g. Deflection Range in ML direction during SAS or walk assessments) and are therefore more difficult to interpret. Since our data were derived from young healthy adults, a generalisation to pathological movements is difficult. If decreased movement amplitude is expected in the disease under study, such as hypokinesia in Parkinson’s disease, this may negatively affect signal accuracy with Kinect especially for ‘noisy” landmarks. However, an evaluation of Kinect V1 in healthy controls and Parkinson’s disease patients with mild-moderate severity did not reveal major differences in accuracy between groups [2]. In contrast, for trunk sway during standing, the RoM may even be expected to increase with different diseases which, accordingly, may even improve accuracy compared to our data in healthy subjects [4]. As a consequence, as has been suggested for the validation of other sensor-based motion analysis solutions [23], testing the accuracy of Kinect V2 in the target populations of clinical application should be considered.

Repeatability is another measure that has to be considered for the interpretation of results, as it impacts on the parameters’ potential to track changes. In this respect, all time parameters, the AP trunk movement during postural transition and knee displacement when walking in place showed excellent reliability in immediate retest. Importantly, repeatability analysis yielded rather similar results for both, Kinect V2 and Vicon. Deflection range of sway during standing, although measurable with high accuracy according to correlation with Vicon, showed lower repeatability than sway velocity, that thus proves more favourable as a parameter to follow up postural disturbance. In contrast, although the accuracy for knee excursion in STEPO is only moderate, this parameter is among those with highest repeatability in agreement with previous findings [2]. Again, also the results of repeatability analysis may be distorted with only small between-subject variance seen for some parameters in healthy subjects. Thus, as observed in other studies [24], repeatability measures may even prove better in patient groups with more diverse motor performance. This may also apply for trunk vectors during short walks in conditions where increased trunk motion during gait is to be expected, such as in multiple sclerosis [14].

The results presented here help to select clinical parameters with potential for further clinical application to be validated in patient groups. While some parameters like walking speed or postural sway velocity are already in use as clinical measures, the clinical meaning of others like the leg parameters from stepping in place still need to be defined. As both time and range of the step-like movements in STEPO showed high repeatability, it will be interesting to explore these parameters as potential surrogates of locomotor stepping. Our results may further guide the design of new assessment tasks and derived clinical parameters using Kinect V2 technology.

## Supporting Information

S1 File Vicon Processing Pipeline. This pipeline is used by the Vicon system for preprocessing of recorded data and includes the set of parameters that can be adjusted in the system.
(PIPELINE)

S1 Appendix Description of Mapping from Vicon to Kinect Landmarks. A description of the processing steps to map the Vicon marker positions of a standard gait model to corresponding Kinect V2 landmark locations.
(PDF)

S2 Appendix Landmark Accuracies for each Task. A detailed overview of the signal accuracies per assessment task. Signal accuracy is described as mean 3D distance of zero shifted signals, Pearson’s correlation coeffcient (r) and Signal to noise ratios (SNR).
(PDF)

S1 Fig Correlation Coefficients (r) of all Joints per Assessment in each Dimension. (TIFF)

S2 Fig Example of Calibration Errors in Kinect Data. (PNG)

S3 Fig Example of Calibration Errors in Vicon Data. (PNG)

S1 Table Detailed Overview of detected Outliers. (PDF)
