# Supplementary material for: Accuracy and Reliability of the Kinect Version 2 for Clinical Measurement of Motor Function
Source: PLoS One. 2016 Nov 18;11(11):e0166532. doi: 10.1371/journal.pone.0166532 (PMC5115766; doi:10.1371/journal.pone.0166532)
Supplement: S1 Appendix — A description of the processing steps to map the Vicon marker positions of a standard gait model to corresponding Kinect V2 landmark locations. (PDF) [file pone.0166532.s002.pdf]

This document includes the detailed processing steps to map the Vicon marker positions of a standard gait model to similar Kinect V2 landmark locations. First, the following processing steps were performed:

1. The units of the Vicon coordination system are converted from mm to m.
2. Y and Z dimension of the Vicon system were exchanged to match the orientation of the Kinect system. The resulting orientation of the coordinate system is X describes the left-right direction, Y the up-down direction and Z the direction towards the point of origin (i.e. the Kinect sensor).
3. The orientation of the X direction is inverted to represent the direction of the Kinect coordinate system.

The following describes the mapping of the Vicon markers to the nearest Kinect landmarks.

Table 1: Mapping of Vicon marker to anatomical landmark from Kinect. If more than one marker name is given, the mean of these markers are used.

Kinect landmark	Vicon marker
FootL	LTOE and LTOE5
FootR	RTOE and RTOE5
AnkleL	LANK
AnkleR	RANK
KneeL	LKNE
KneeR	RKNE
HipL	LASI
HipR	RASI
Spine base	RASI,LASI,LPSI and RPSI
Spine mid	CLAV and Spine base
Spine shoulder	C7
Head	RFHD, LFHD, RBHD and LBHD
ShoulderL	LSHO
ShoulderR	RSHO
ElbowL	LELB
ElbowR	RELB
WristL	LWRA and LWRB
WristR	RWRA and RWRB
HandL	LFIN
HandR	RFIN
