# Supplementary material for: Accuracy and Reliability of the Kinect Version 2 for Clinical Measurement of Motor Function
Source: PLoS One. 2016 Nov 18;11(11):e0166532. doi: 10.1371/journal.pone.0166532 (PMC5115766; doi:10.1371/journal.pone.0166532)
Supplement: S2 Appendix — A detailed overview of the signal accuracies per assessment task. Signal accuracy is described as mean 3D distance of zero shifted signals, Pearson’s correlation coeffcient (r) and Signal to noise ratios (SNR). (PDF) [file pone.0166532.s003.pdf]

This document contains a detailed overview of the signal accuracies per assessment task. Signal accuracy is described as mean 3D distance of zero shifted signals, Pearson's correlation coefficient ( $r$) and Signal to noise ratios (SNR).

| Joint name | 3D 3D Diff | $r$ ML | $r$ V | $r$ AP | SNR ML | SNR V | SNR AP |
|---|---|---|---|---|---|---|---|
| ankleL | 0.02 (0.01) | 0.61 (0.30) | -0.02 (0.48) | 0.62 (0.29) | -5.43 (5.04) | -20.99 (6.04) | -7.09 (4.19) |
| ankleR | 0.02 (0.01) | 0.60 (0.30) | -0.01 (0.45) | 0.74 (0.28) | -4.00 (5.97) | -20.60 (6.71) | -7.13 (5.53) |
| elbowL | 0.02 (0.01) | 0.89 (0.21) | 1.00 (0.00) | 1.00 (0.01) | 8.31 (4.60) | 21.08 (3.17) | 23.37 (3.87) |
| elbowR | 0.02 (0.01) | 0.90 (0.20) | 1.00 (0.01) | 1.00 (0.01) | 8.32 (4.74) | 21.54 (3.28) | 23.18 (3.59) |
| footL | 0.04 (0.02) | 0.22 (0.33) | -0.56 (0.24) | 0.10 (0.26) | -15.53 (6.37) | -27.54 (4.71) | -28.66 (7.86) |
| footR | 0.04 (0.01) | 0.30 (0.37) | -0.52 (0.26) | 0.23 (0.30) | -14.12 (6.05) | -25.68 (4.02) | -28.25 (8.48) |
| handL | 0.02 (0.01) | 0.85 (0.19) | 0.99 (0.01) | 0.99 (0.01) | 7.36 (4.74) | 19.75 (3.70) | 20.98 (3.96) |
| handR | 0.02 (0.01) | 0.80 (0.26) | 0.99 (0.01) | 0.99 (0.02) | 6.62 (6.08) | 19.06 (3.71) | 20.83 (4.26) |
| head | 0.02 (0.01) | 0.87 (0.29) | 1.00 (0.01) | 0.99 (0.02) | 8.57 (5.57) | 21.87 (3.54) | 23.00 (4.75) |
| hipL | 0.02 (0.01) | 0.63 (0.34) | 0.99 (0.01) | 0.99 (0.01) | 0.59 (4.58) | 19.24 (4.08) | 19.78 (3.90) |
| hipR | 0.02 (0.01) | 0.57 (0.41) | 1.00 (0.01) | 0.99 (0.01) | -0.66 (4.75) | 19.37 (3.60) | 20.02 (3.69) |
| kneeL | 0.03 (0.01) | 0.76 (0.29) | 0.43 (0.39) | 0.50 (0.47) | 5.02 (5.10) | -6.79 (5.35) | 1.97 (5.06) |
| kneeR | 0.03 (0.01) | 0.84 (0.26) | 0.44 (0.36) | 0.53 (0.47) | 6.97 (6.12) | -6.73 (5.45) | 2.36 (4.95) |
| neck | 0.02 (0.01) | 0.85 (0.23) | 0.99 (0.01) | 0.99 (0.02) | 6.83 (5.25) | 17.42 (2.63) | 21.94 (4.04) |
| shoulderL | 0.02 (0.01) | 0.83 (0.25) | 1.00 (0.01) | 0.99 (0.02) | 5.30 (4.80) | 23.77 (4.00) | 18.84 (2.90) |
| shoulderR | 0.02 (0.01) | 0.87 (0.23) | 1.00 (0.01) | 0.99 (0.01) | 6.95 (4.57) | 23.99 (4.07) | 18.37 (2.46) |
| spineBase | 0.02 (0.01) | 0.64 (0.35) | 0.99 (0.01) | 0.99 (0.01) | 0.37 (4.79) | 18.87 (3.41) | 19.41 (3.96) |
| spineMid | 0.02 (0.01) | 0.79 (0.32) | 1.00 (0.01) | 1.00 (0.01) | 5.18 (5.46) | 22.57 (4.00) | 23.73 (4.74) |
| spineShoulder | 0.02 (0.01) | 0.85 (0.27) | 1.00 (0.01) | 1.00 (0.01) | 7.16 (5.20) | 21.50 (3.33) | 23.54 (4.47) |
| wristL | 0.02 (0.01) | 0.84 (0.20) | 1.00 (0.00) | 1.00 (0.01) | 7.00 (4.57) | 21.02 (3.29) | 22.27 (4.00) |
| wristR | 0.02 (0.01) | 0.77 (0.27) | 1.00 (0.01) | 0.99 (0.01) | 5.20 (6.17) | 20.87 (3.94) | 21.21 (4.04) |

Table 1: SAS

| Joint name | 3D Diff | $r$ ML | $r$ V | $r$ AP | SNR ML | SNR V | SNR AP |
|---|---|---|---|---|---|---|---|
| ankleL | 0.07 (0.02) | 0.71 (0.30) | 0.53 (0.21) | 1.00 (0.00) | 0.10 (5.44) | 1.50 (1.26) | 24.50 (5.39) |
| ankleR | 0.08 (0.03) | 0.63 (0.31) | 0.43 (0.22) | 0.99 (0.01) | -1.56 (4.81) | 0.82 (1.51) | 20.40 (4.81) |
| elbowL | 0.03 (0.00) | 0.86 (0.12) | 0.38 (0.30) | 1.00 (0.00) | 5.10 (3.18) | -5.28 (3.15) | 38.39 (2.35) |
| elbowR | 0.02 (0.00) | 0.85 (0.13) | 0.47 (0.30) | 1.00 (0.00) | 4.41 (3.26) | -2.37 (3.05) | 39.49 (2.56) |
| footL | 0.09 (0.02) | 0.60 (0.28) | 0.19 (0.18) | 0.99 (0.01) | -1.95 (4.57) | -4.36 (1.62) | 19.41 (3.17) |
| footR | 0.10 (0.03) | 0.56 (0.28) | 0.14 (0.21) | 0.99 (0.01) | -2.77 (3.85) | -6.67 (2.84) | 17.69 (2.93) |
| handL | 0.03 (0.01) | 0.86 (0.13) | 0.90 (0.10) | 1.00 (0.00) | 4.70 (4.26) | 7.34 (2.54) | 33.77 (4.40) |
| handR | 0.03 (0.01) | 0.84 (0.18) | 0.86 (0.10) | 1.00 (0.00) | 4.85 (4.75) | 6.54 (3.27) | 36.43 (4.26) |
| head | 0.02 (0.00) | 0.86 (0.16) | 0.80 (0.16) | 1.00 (0.00) | 3.63 (3.99) | 4.03 (4.92) | 43.29 (1.33) |
| hipL | 0.02 (0.00) | 0.83 (0.16) | 0.71 (0.15) | 1.00 (0.00) | 1.78 (3.39) | 1.82 (3.49) | 36.02 (2.70) |
| hipR | 0.02 (0.00) | 0.83 (0.16) | 0.71 (0.15) | 1.00 (0.00) | 2.06 (3.34) | 2.19 (3.63) | 34.87 (1.99) |
| kneeL | 0.04 (0.01) | 0.76 (0.20) | 0.20 (0.21) | 1.00 (0.00) | 0.58 (3.68) | -4.06 (1.57) | 30.24 (2.38) |
| kneeR | 0.04 (0.01) | 0.73 (0.25) | 0.12 (0.23) | 1.00 (0.00) | 0.37 (3.97) | -4.25 (1.75) | 28.94 (2.36) |
| neck | 0.02 (0.00) | 0.85 (0.14) | 0.80 (0.19) | 1.00 (0.00) | 3.89 (3.53) | 5.01 (5.27) | 42.85 (0.82) |
| shoulderL | 0.02 (0.00) | 0.83 (0.18) | 0.81 (0.14) | 1.00 (0.00) | 2.47 (4.31) | 4.76 (3.92) | 40.59 (1.61) |
| shoulderR | 0.02 (0.00) | 0.87 (0.15) | 0.82 (0.12) | 1.00 (0.00) | 3.63 (4.72) | 4.60 (3.73) | 40.61 (1.71) |
| spineBase | 0.02 (0.00) | 0.79 (0.19) | 0.79 (0.13) | 1.00 (0.00) | 0.72 (3.71) | 2.92 (4.61) | 39.14 (1.97) |
| spineMid | 0.02 (0.00) | 0.82 (0.18) | 0.83 (0.13) | 1.00 (0.00) | 1.23 (3.87) | 5.02 (4.90) | 42.69 (1.42) |
| spineShoulder | 0.02 (0.00) | 0.82 (0.19) | 0.83 (0.14) | 1.00 (0.00) | 1.24 (4.24) | 5.32 (4.77) | 43.31 (0.80) |
| wristL | 0.03 (0.01) | 0.85 (0.15) | 0.93 (0.06) | 1.00 (0.00) | 3.95 (3.86) | 8.02 (3.37) | 34.30 (3.74) |
| wristR | 0.02 (0.00) | 0.84 (0.18) | 0.89 (0.08) | 1.00 (0.00) | 3.63 (3.81) | 6.00 (3.62) | 35.84 (3.06) |

Table 2: SWCS

| Joint name | 3D Diff | $r$ ML | $r$ V | $r$ AP | SNR ML | SNR V | SNR AP |
|---|---|---|---|---|---|---|---|
| ankleL | 0.08 (0.04) | 0.72 (0.34) | 0.55 (0.19) | 1.00 (0.01) | 1.97 (5.76) | 1.60 (1.54) | 22.59 (5.16) |
| ankleR | 0.10 (0.04) | 0.67 (0.32) | 0.38 (0.27) | 0.99 (0.01) | 0.10 (5.57) | 0.28 (1.61) | 19.18 (4.51) |
| elbowL | 0.03 (0.01) | 0.79 (0.21) | 0.16 (0.34) | 1.00 (0.00) | 3.14 (3.99) | -3.42 (3.83) | 34.84 (3.18) |
| elbowR | 0.03 (0.01) | 0.76 (0.24) | 0.29 (0.33) | 1.00 (0.00) | 1.83 (4.51) | -0.95 (2.85) | 35.58 (2.64) |
| footL | 0.10 (0.04) | 0.65 (0.32) | 0.15 (0.22) | 0.99 (0.01) | -0.38 (5.14) | -4.80 (2.40) | 17.80 (2.94) |
| footR | 0.12 (0.04) | 0.62 (0.31) | 0.13 (0.24) | 0.99 (0.01) | -1.24 (5.11) | -6.44 (2.79) | 16.32 (2.85) |
| handL | 0.04 (0.01) | 0.91 (0.12) | 0.93 (0.06) | 1.00 (0.00) | 8.39 (4.51) | 9.04 (3.26) | 30.78 (4.27) |
| handR | 0.03 (0.01) | 0.91 (0.11) | 0.87 (0.18) | 1.00 (0.00) | 8.74 (5.44) | 7.64 (4.10) | 32.15 (4.41) |
| head | 0.02 (0.00) | 0.88 (0.17) | 0.76 (0.20) | 1.00 (0.00) | 3.61 (4.12) | 2.77 (5.51) | 43.22 (2.17) |
| hipL | 0.03 (0.01) | 0.80 (0.20) | 0.58 (0.29) | 1.00 (0.00) | 1.99 (3.69) | 1.83 (3.88) | 32.97 (2.96) |
| hipR | 0.03 (0.01) | 0.81 (0.19) | 0.63 (0.31) | 1.00 (0.00) | 2.16 (3.77) | 2.74 (4.08) | 32.39 (2.50) |
| kneeL | 0.05 (0.01) | 0.76 (0.24) | 0.03 (0.28) | 1.00 (0.00) | 2.05 (4.83) | -3.13 (2.06) | 26.38 (3.22) |
| kneeR | 0.06 (0.02) | 0.76 (0.25) | 0.00 (0.26) | 1.00 (0.00) | 2.28 (4.32) | -3.30 (1.90) | 25.90 (2.76) |
| neck | 0.02 (0.00) | 0.82 (0.20) | 0.66 (0.27) | 1.00 (0.00) | 3.06 (4.52) | 2.22 (4.81) | 42.54 (2.02) |
| shoulderL | 0.02 (0.01) | 0.79 (0.30) | 0.75 (0.24) | 1.00 (0.00) | 1.96 (4.88) | 4.11 (4.65) | 37.78 (2.24) |
| shoulderR | 0.02 (0.01) | 0.84 (0.22) | 0.76 (0.25) | 1.00 (0.00) | 3.01 (5.51) | 3.97 (4.76) | 38.16 (2.37) |
| spineBase | 0.02 (0.01) | 0.77 (0.28) | 0.75 (0.20) | 1.00 (0.00) | -0.63 (4.89) | 3.62 (4.70) | 38.34 (2.51) |
| spineMid | 0.02 (0.00) | 0.74 (0.33) | 0.78 (0.21) | 1.00 (0.00) | -0.19 (5.11) | 4.84 (4.86) | 43.01 (2.22) |
| spineShoulder | 0.02 (0.00) | 0.70 (0.33) | 0.80 (0.23) | 1.00 (0.00) | 0.42 (4.88) | 5.40 (5.37) | 42.89 (1.74) |
| wristL | 0.03 (0.01) | 0.89 (0.16) | 0.93 (0.07) | 1.00 (0.00) | 7.06 (4.48) | 8.41 (3.80) | 31.44 (4.24) |
| wristR | 0.03 (0.01) | 0.90 (0.14) | 0.86 (0.14) | 1.00 (0.00) | 7.13 (5.52) | 6.26 (4.62) | 32.39 (3.55) |

Table 3: SWMS

| Joint name | 3D Diff | $r$ ML | $r$ V | $r$ AP | SNR ML | SNR V | SNR AP |
|---|---|---|---|---|---|---|---|
| ankleL | 0.06 (0.02) | 0.74 (0.12) | 0.15 (0.21) | 1.00 (0.00) | 2.78 (2.86) | -1.98 (2.55) | 24.69 (2.48) |
| ankleR | 0.06 (0.01) | 0.87 (0.09) | 0.10 (0.23) | 1.00 (0.00) | 6.88 (3.44) | -2.37 (2.58) | 24.58 (2.48) |
| elbowL | 0.02 (0.00) | 0.89 (0.10) | 0.51 (0.24) | 1.00 (0.00) | 5.16 (3.82) | -2.10 (4.14) | 38.93 (3.14) |
| elbowR | 0.02 (0.01) | 0.82 (0.18) | 0.51 (0.26) | 1.00 (0.00) | 5.28 (5.30) | -1.50 (4.49) | 38.90 (3.48) |
| footL | 0.07 (0.02) | 0.67 (0.13) | -0.05 (0.16) | 1.00 (0.00) | 1.27 (2.40) | -8.39 (3.71) | 21.72 (1.57) |
| footR | 0.08 (0.02) | 0.80 (0.12) | -0.07 (0.15) | 1.00 (0.00) | 4.56 (2.79) | -9.17 (3.58) | 21.63 (1.72) |
| handL | 0.02 (0.01) | 0.94 (0.08) | 0.69 (0.20) | 1.00 (0.00) | 8.46 (4.58) | 1.76 (4.35) | 35.10 (3.63) |
| handR | 0.02 (0.01) | 0.84 (0.15) | 0.69 (0.21) | 1.00 (0.00) | 4.91 (5.60) | 2.05 (4.57) | 35.59 (4.04) |
| head | 0.02 (0.01) | 0.92 (0.07) | 0.58 (0.42) | 1.00 (0.00) | 7.20 (4.11) | 1.97 (5.38) | 40.10 (2.60) |
| hipL | 0.02 (0.00) | 0.85 (0.10) | 0.50 (0.32) | 1.00 (0.00) | 3.00 (3.50) | -1.75 (3.51) | 37.91 (2.13) |
| hipR | 0.02 (0.00) | 0.85 (0.11) | 0.45 (0.35) | 1.00 (0.00) | 3.33 (3.55) | -2.12 (3.83) | 37.66 (2.25) |
| kneeL | 0.04 (0.01) | 0.79 (0.09) | 0.21 (0.20) | 1.00 (0.00) | 2.75 (2.04) | -4.64 (2.01) | 28.05 (2.06) |
| kneeR | 0.04 (0.01) | 0.86 (0.08) | 0.25 (0.16) | 1.00 (0.00) | 5.09 (2.76) | -4.64 (1.79) | 28.67 (1.97) |
| neck | 0.02 (0.00) | 0.90 (0.08) | 0.07 (0.43) | 1.00 (0.00) | 6.44 (3.51) | -7.20 (4.87) | 37.64 (3.36) |
| shoulderL | 0.02 (0.00) | 0.90 (0.07) | 0.51 (0.38) | 1.00 (0.00) | 5.89 (3.71) | 0.16 (4.20) | 40.21 (2.08) |
| shoulderR | 0.02 (0.00) | 0.89 (0.11) | 0.53 (0.32) | 1.00 (0.00) | 7.11 (4.49) | -0.81 (4.99) | 39.99 (2.49) |
| spineBase | 0.02 (0.00) | 0.83 (0.14) | 0.51 (0.44) | 1.00 (0.00) | 2.46 (3.57) | -3.68 (4.98) | 39.74 (2.60) |
| spineMid | 0.02 (0.00) | 0.88 (0.10) | 0.38 (0.50) | 1.00 (0.00) | 3.94 (3.62) | -2.26 (5.30) | 38.55 (3.32) |
| spineShoulder | 0.02 (0.01) | 0.90 (0.08) | 0.22 (0.46) | 1.00 (0.00) | 5.39 (3.87) | -3.29 (4.56) | 36.86 (3.45) |
| wristL | 0.02 (0.01) | 0.93 (0.08) | 0.66 (0.21) | 1.00 (0.00) | 7.14 (4.77) | 0.20 (4.26) | 36.45 (2.78) |
| wristR | 0.02 (0.00) | 0.83 (0.16) | 0.63 (0.24) | 1.00 (0.00) | 4.16 (4.90) | 0.74 (4.88) | 37.03 (2.96) |

Table 4: SWL

| Joint name | 3D Diff | $r$ ML | $r$ V | $r$ AP | SNR ML | SNR V | SNR AP |
|---|---|---|---|---|---|---|---|
| ankleL | 0.01 (0.01) | 0.35 (0.36) | -0.03 (0.32) | 0.21 (0.30) | -16.01 (9.02) | -21.76 (7.58) | -15.34 (8.25) |
| ankleR | 0.01 (0.01) | 0.32 (0.31) | -0.05 (0.29) | 0.22 (0.32) | -18.05 (8.92) | -26.22 (8.91) | -14.79 (6.17) |
| elbowL | 0.00 (0.00) | 0.96 (0.11) | 0.39 (0.31) | 0.97 (0.03) | 12.82 (3.65) | -1.77 (4.13) | 13.58 (3.47) |
| elbowR | 0.00 (0.00) | 0.96 (0.10) | 0.40 (0.29) | 0.97 (0.06) | 12.36 (3.58) | -1.29 (3.43) | 13.90 (3.51) |
| footL | 0.01 (0.01) | 0.12 (0.28) | -0.06 (0.21) | 0.05 (0.23) | -24.96 (8.80) | -37.05 (9.17) | -36.33 (12.87) |
| footR | 0.03 (0.02) | 0.10 (0.26) | 0.01 (0.22) | 0.04 (0.23) | -30.46 (8.87) | -41.88 (9.54) | -40.92 (14.06) |
| handL | 0.00 (0.00) | 0.88 (0.12) | 0.37 (0.25) | 0.88 (0.08) | 6.72 (2.74) | -3.23 (4.34) | 7.11 (3.33) |
| handR | 0.00 (0.00) | 0.89 (0.11) | 0.31 (0.31) | 0.88 (0.08) | 7.09 (3.09) | -4.74 (5.18) | 7.39 (3.54) |
| head | 0.00 (0.00) | 0.96 (0.11) | 0.60 (0.32) | 0.97 (0.06) | 13.06 (3.50) | -4.15 (5.62) | 14.05 (3.76) |
| hipL | 0.00 (0.00) | 0.92 (0.12) | 0.26 (0.39) | 0.97 (0.03) | 9.34 (3.19) | -1.90 (3.43) | 11.89 (2.79) |
| hipR | 0.00 (0.00) | 0.93 (0.11) | 0.44 (0.40) | 0.96 (0.05) | 9.47 (3.11) | 0.07 (3.81) | 11.38 (2.79) |
| kneeL | 0.00 (0.00) | 0.83 (0.15) | -0.30 (0.32) | 0.85 (0.12) | 2.28 (4.04) | -13.04 (5.04) | 4.84 (3.89) |
| kneeR | 0.00 (0.00) | 0.82 (0.16) | -0.26 (0.35) | 0.78 (0.19) | 2.95 (3.76) | -13.77 (5.76) | 3.13 (5.48) |
| neck | 0.00 (0.00) | 0.95 (0.10) | -0.19 (0.40) | 0.98 (0.03) | 11.89 (3.67) | -6.23 (3.64) | 16.00 (3.74) |
| shoulderL | 0.00 (0.00) | 0.96 (0.10) | 0.66 (0.20) | 0.97 (0.04) | 12.78 (3.45) | 2.32 (2.89) | 12.89 (3.54) |
| shoulderR | 0.00 (0.00) | 0.95 (0.11) | 0.59 (0.21) | 0.96 (0.08) | 11.09 (3.40) | -0.18 (2.97) | 12.32 (3.86) |
| spineBase | 0.00 (0.00) | 0.92 (0.11) | 0.34 (0.40) | 0.97 (0.04) | 8.85 (2.79) | -8.10 (4.16) | 12.46 (2.78) |
| spineMid | 0.00 (0.00) | 0.96 (0.10) | 0.55 (0.36) | 0.98 (0.03) | 12.14 (3.42) | 0.22 (4.26) | 15.01 (3.33) |
| spineShoulder | 0.00 (0.00) | 0.95 (0.10) | 0.59 (0.32) | 0.98 (0.03) | 12.22 (4.08) | 2.24 (3.76) | 14.74 (3.62) |
| wristL | 0.00 (0.00) | 0.93 (0.12) | 0.48 (0.31) | 0.95 (0.05) | 10.12 (3.17) | -0.98 (4.08) | 11.13 (3.53) |
| wristR | 0.00 (0.00) | 0.93 (0.10) | 0.51 (0.28) | 0.95 (0.07) | 9.70 (3.48) | -2.17 (5.72) | 11.11 (3.66) |

Table 5: SOCE

| Joint name | 3D Diff | $r$ ML | $r$ V | $r$ AP | SNR ML | SNR V | SNR AP |
|---|---|---|---|---|---|---|---|
| ankleL | 0.05 (0.01) | 0.85 (0.11) | 0.74 (0.22) | 0.92 (0.12) | 5.55 (3.43) | 3.09 (1.68) | 10.50 (5.92) |
| ankleR | 0.05 (0.01) | 0.88 (0.07) | 0.66 (0.26) | 0.94 (0.07) | 7.00 (3.08) | 2.37 (1.66) | 11.20 (5.45) |
| elbowL | 0.02 (0.01) | 0.95 (0.05) | 0.26 (0.35) | 0.98 (0.02) | 11.15 (3.52) | -3.15 (3.81) | 15.53 (5.15) |
| elbowR | 0.02 (0.01) | 0.95 (0.06) | 0.34 (0.38) | 0.98 (0.03) | 11.03 (4.08) | -1.32 (3.48) | 15.70 (5.61) |
| footL | 0.05 (0.01) | 0.63 (0.21) | 0.37 (0.41) | 0.75 (0.27) | 0.37 (3.63) | 0.92 (2.93) | 3.92 (5.60) |
| footR | 0.05 (0.01) | 0.64 (0.22) | 0.22 (0.44) | 0.76 (0.27) | -0.27 (4.03) | 0.08 (2.91) | 4.04 (6.13) |
| handL | 0.05 (0.03) | 0.91 (0.05) | 0.72 (0.15) | 0.94 (0.04) | 7.95 (2.35) | 3.13 (2.58) | 10.95 (4.39) |
| handR | 0.05 (0.04) | 0.91 (0.06) | 0.73 (0.16) | 0.95 (0.04) | 8.07 (2.49) | 3.79 (3.15) | 11.84 (4.85) |
| head | 0.01 (0.00) | 0.98 (0.02) | 0.64 (0.20) | 1.00 (0.01) | 14.02 (3.75) | 0.39 (4.02) | 23.88 (6.65) |
| hipL | 0.01 (0.00) | 0.95 (0.06) | 0.60 (0.19) | 0.98 (0.02) | 10.81 (3.57) | 0.85 (2.48) | 17.52 (5.81) |
| hipR | 0.01 (0.00) | 0.95 (0.05) | 0.62 (0.27) | 0.98 (0.02) | 10.67 (3.38) | 1.31 (3.35) | 18.11 (6.16) |
| kneeL | 0.04 (0.01) | 0.86 (0.09) | 0.81 (0.13) | 0.95 (0.03) | 5.95 (3.59) | 4.58 (2.32) | 10.16 (2.50) |
| kneeR | 0.04 (0.01) | 0.85 (0.11) | 0.82 (0.07) | 0.95 (0.03) | 5.47 (3.19) | 4.57 (2.08) | 10.03 (2.36) |
| neck | 0.01 (0.00) | 0.98 (0.02) | 0.33 (0.24) | 1.00 (0.01) | 11.84 (2.47) | -1.06 (2.28) | 25.58 (5.58) |
| shoulderL | 0.01 (0.00) | 0.99 (0.01) | 0.74 (0.22) | 0.99 (0.01) | 15.22 (2.73) | 3.92 (2.50) | 21.24 (5.49) |
| shoulderR | 0.01 (0.00) | 0.98 (0.02) | 0.62 (0.23) | 0.99 (0.01) | 14.03 (3.12) | 2.42 (2.46) | 20.39 (5.07) |
| spineBase | 0.01 (0.00) | 0.95 (0.05) | 0.59 (0.15) | 0.99 (0.01) | 11.00 (3.40) | -0.87 (2.80) | 20.47 (5.84) |
| spineMid | 0.01 (0.00) | 0.98 (0.02) | 0.66 (0.17) | 0.99 (0.01) | 14.35 (3.02) | 1.39 (2.64) | 23.07 (5.88) |
| spineShoulder | 0.01 (0.00) | 0.98 (0.01) | 0.64 (0.18) | 1.00 (0.01) | 14.48 (2.62) | 1.93 (2.61) | 24.26 (5.86) |
| wristL | 0.03 (0.03) | 0.94 (0.05) | 0.84 (0.09) | 0.96 (0.04) | 10.14 (3.46) | 5.53 (2.57) | 13.02 (4.91) |
| wristR | 0.03 (0.03) | 0.93 (0.06) | 0.81 (0.13) | 0.96 (0.04) | 10.30 (3.80) | 5.02 (3.59) | 14.04 (5.57) |

Table 6: STEPO
