# Supplementary figures and images for: Accuracy and Reliability of the Kinect Version 2 for Clinical Measurement of Motor Function
Source: PLoS One. 2016 Nov 18;11(11):e0166532. doi: 10.1371/journal.pone.0166532 (PMC5115766; doi:10.1371/journal.pone.0166532)

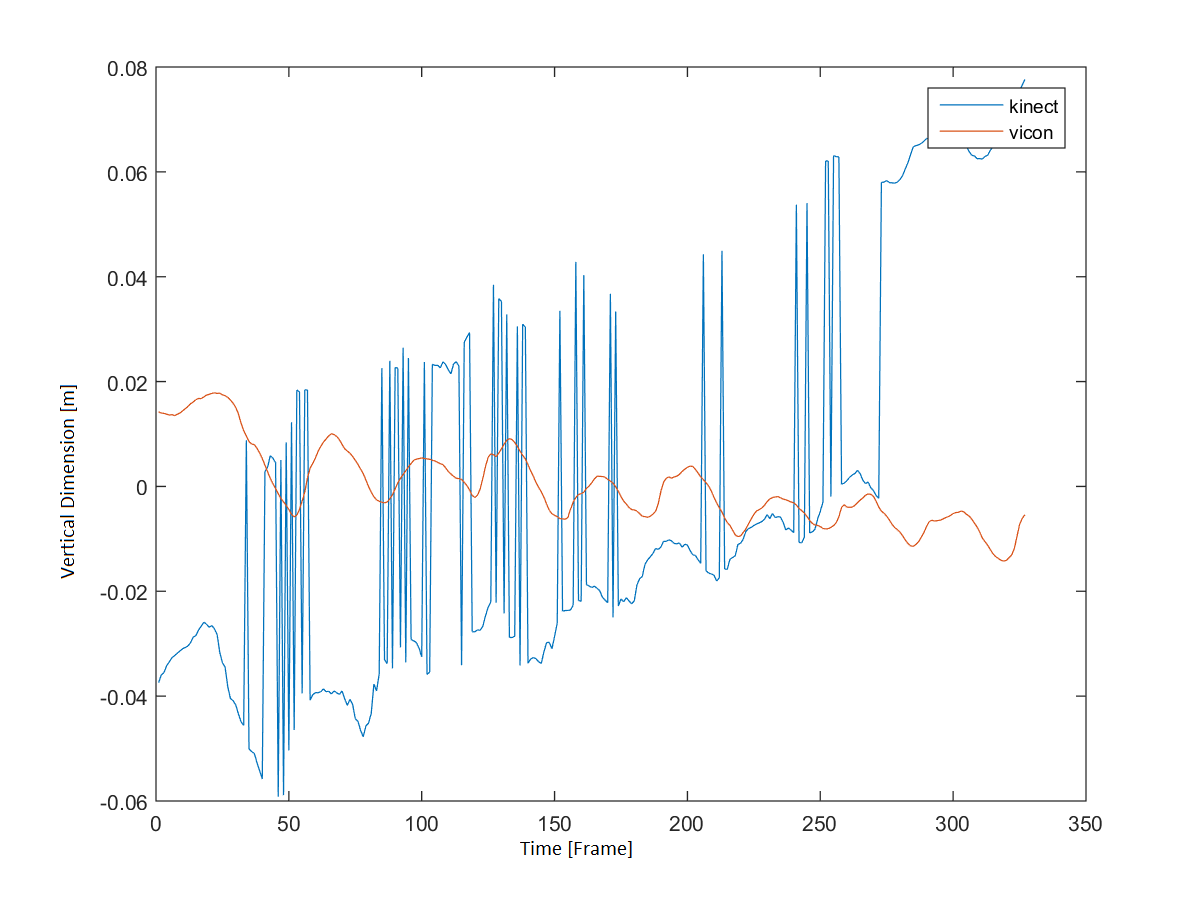

Supplement: S2 Fig — (PNG) [file pone.0166532.s005.png]

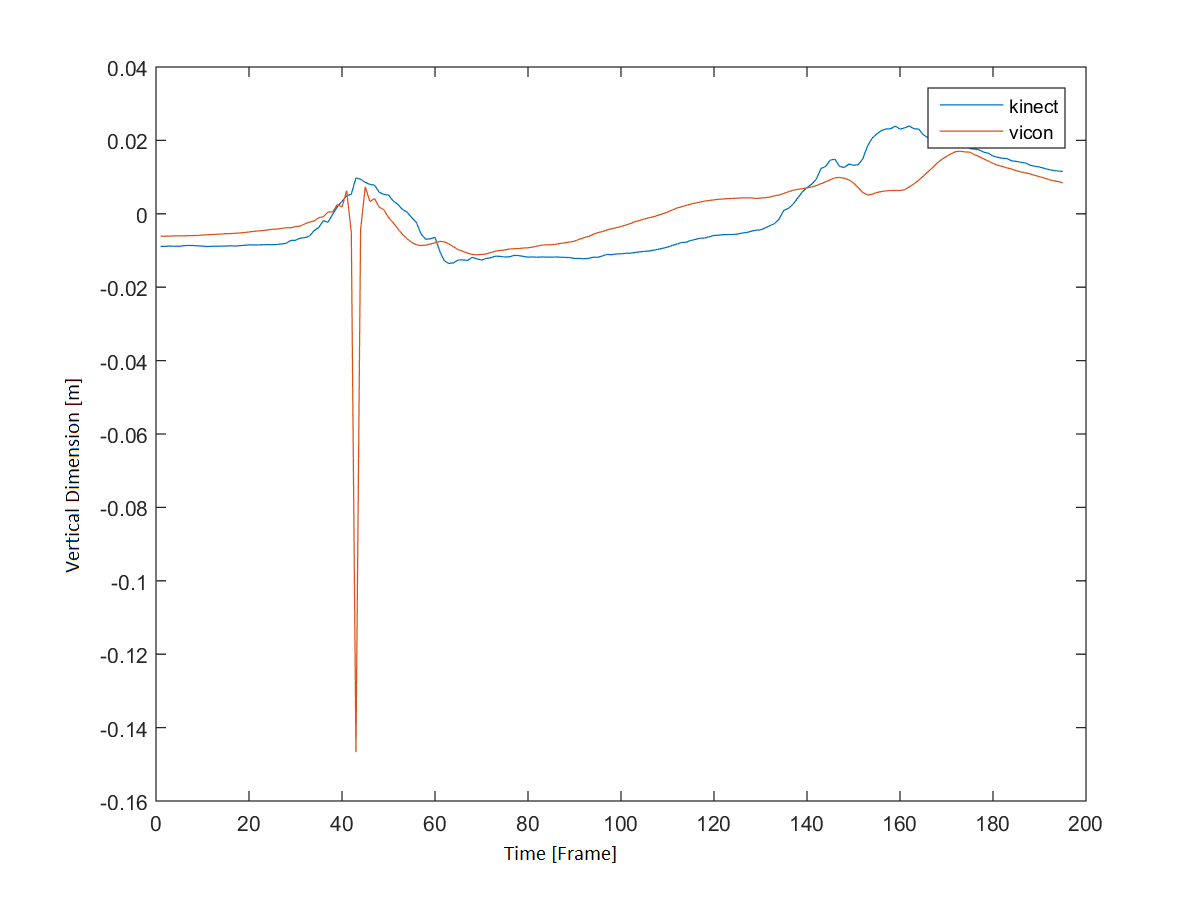

Supplement: S3 Fig — (PNG) [file pone.0166532.s006.png]
