# Supplementary material for: Accuracy and Reliability of the Kinect Version 2 for Clinical Measurement of Motor Function
Source: PLoS One. 2016 Nov 18;11(11):e0166532. doi: 10.1371/journal.pone.0166532 (PMC5115766; doi:10.1371/journal.pone.0166532)
Supplement: S1 Table — (PDF) [file pone.0166532.s007.pdf]

This document includes the detected outliers, that are excluded from parameter extraction and analyses.

JointName	Repetition	SubjectId	AssessmentId	Dimension	SNR	Max. Speed	Amplitude Difference
ankleR	4	11	SAS	Y	-12.88	0.08	0.12
spineShoulder	1	8	SLW	Y	-15.16	0.06	0.10
spineShoulder	2	8	SLW	Y	-13.59	0.06	0.10
ankleR	3	9	POCO	Y	-29.51	0.10	0.11
ankleL	4	11	POCO	Y	-46.42	1.21	1.24
ankleR	4	11	POCO	Y	-54.18	1.29	1.29
spineShoulder	4	11	POCO	Y	-15.05	0.14	0.14
ankleL	1	14	POCO	Y	-35.09	0.13	0.23
ankleL	2	14	POCO	Y	-36.71	0.16	0.24
ankleL	3	14	POCO	Y	-15.41	0.13	0.14
ankleL	4	14	POCO	Y	-37.23	0.14	0.21
ankleL	5	14	POCO	Y	-28.45	0.14	0.18
ankleL	5	15	POCO	Y	-23.64	0.08	0.10
ankleR	5	19	POCO	Y	-33.69	0.09	0.17
ankleL	4	11	POCO	X	-50.05	2.09	2.10
ankleR	4	11	POCO	X	-53.45	2.02	2.02
spineShoulder	4	11	POCO	X	-28.98	2.04	2.04
ankleL	4	11	POCO	Z	-58.39	3.31	3.32
ankleR	4	11	POCO	Z	-61.38	3.30	3.31
spineShoulder	4	11	POCO	Z	-29.87	2.08	2.07
ankleL	1	14	POCO	Z	-26.81	0.06	0.12
ankleL	4	14	POCO	Z	-26.08	0.06	0.11

Table 1: Overview of excluded Outliers based on Kinect Data

JointName	Repetition	SubjectId	AssessmentId	Dimension	SNR	Max. Speed	Amplitude Difference
spineShoulder	1	1	SAS	Y	17.54	0.065	-0.047

Table 2: Overview of excluded Outliers based on Vicon Data
